# National Survey of Radiotherapy Utilization Trends from 2015 to 2019, Based on the National Database of Health Insurance Claims and Specific Health Checkups of Japan

**DOI:** 10.31662/jmaj.2022-0142

**Published:** 2023-05-12

**Authors:** Atsuto Katano, Masanari Minamitani, Hideomi Yamashita, Keiichi Nakagawa

**Affiliations:** 1 Department of Radiology, The University of Tokyo Hospital, Tokyo, Japan; 2 Department of Comprehensive Radiation Oncology, The University of Tokyo, Tokyo, Japan

**Keywords:** radiotherapy, cancer, treatment

Japan achieved a universal health coverage insurance system, and medical costs are paid by medical insurers depending on the patient’s insurance program, which is either the national health insurance or employees’health insurance ^(1), (2), (3)^. Receipts are statements of medical fees, issued by medical institutions to medical insurers, to document the covered medical services. During the medical care system reform in 2006, the medical cost optimization plan was introduced to establish a system that promoted the optimization of medical costs from medium to long term. In 2009, the Ministry of Health, Labour and Welfare (MHLW) of Japan initiated the National Database of Health Insurance Claims and Specific Health Checkups of Japan (NDB) to prepare, implement, and evaluate the medical cost optimization plan. Information on receipts and specified health examinations was collected. Since October 2016, the MHLW has permitted the secondary use of a part of NDB as a public database (NDB open data Japan), which made health care more accessible to the general public. Although not all the receipts for insured treatments were stored in NDB, the covered ratio of digitally electronic receipts in the medical field reached over 98% ^(4)^.

Radiotherapy plays an important role in cancer treatment ^(5)^. It is a curative and palliative treatment option that relieves cancer symptoms. Radiotherapy has been used in conjunction with other treatment strategies, such as preoperative and postoperative adjuvant therapy and chemotherapy. It has been more than 100 years since radiation was first used as a means of treating cancer. Over time, radiotherapy has advanced rapidly with the development of radiotherapy equipment, radiobiology, and computational planning systems ^(6)^. Radiotherapy methods have been further developed to facilitate the delivery of high doses of radiation to cancer cells while minimizing radiation exposure to surrounding normal tissues. This development achieves high therapeutic efficacy with minimal side effects.

The demand for radiotherapy in Japan is expected to increase due to the rising incidence of cancer and aging of patients with cancer. However, there are extremely limited methods to investigate the current utilization of radiotherapy practice in Japan. In this study, we aimed to conduct an analysis using a novel method, NDB open data Japan.

Data on the number of receipts from 2015 to 2019 were collected from NDB open data Japan. It was extracted using the medical fee code associated with radiotherapy. This study was exempt from institutional review board approval because it used anonymized public open data only and did not contain personally identifiable information.

The radiotherapy management fee (Medical fee code: M000) was applied to conventional external beam radiotherapy, intensity-modulated radiation therapy, and brachytherapy. This fee was calculated once per radiation treatment plan. When the treatment plan was changed during the radiotherapy period, such as in boost planning, the fee was calculated up to a maximum of one additional fee. The radioisotope therapy management fee (Medical fee code: M000-2) was applied to unsealed internal radiation therapy, containing radioactive materials, which were administered intravenously or orally. The medical fee code from M001-2 to M003 was applied only once in a patient undergoing gamma knife radiosurgery; stereotactic body radiotherapy; and particle and total body irradiation and hyperthermia therapies. Since 2016, gamma knife radiosurgery could alternatively be managed as a short-stay surgery basic fee (Medical fee code: A400).

Software program R was used for statistical comparison. Chi-squared test was used to determine statistical difference. For statistical significance, p < 0.05 was set as the threshold value.

The total number of the receipts, associated with radiotherapy, slightly increased with a compound annual growth rate (CAGR) of 2.13%. CAGR measures an annual growth rate over a period, which was defined as geometric mean per year obtained from the growth rate over multiple years. In 2019, there were 321,788 receipts, including 145,093 (45%) and 176,695 (55%) receipts from inpatients and outpatients, respectively (Table 1).

**Table 1.** The Number of Receipts Associated with Radiotherapy in 2015, 2017, and 2019.

Medical fee code	Category name	Description	2015	2017	2019	CAGR******* (%)
M000	Radiotherapy management fee	Simple^*^	61212	53932	49984	−4.94
		Complex^**^	67798	61621	60276	−2.90
		Special^***^	93785	98829	108337	3.67
		IMRT^****^	23454	32123	41894	15.61
M000-2	Radioisotope therapy management fee	Thyroid cancer	9482	9134	8996	−1.31
		Solid tumor bone metastases	1968	3438	292	−37.94
		Non-Hodgkin lymphoma (B cell)	364	513	340	−1.69
		CRPC^*****^ with bone metastases	0	0	4658	N.A.
M001-2	Stereotactic radiotherapy with gamma knife	SRT^******^ with gamma knife	13371	3424	11008	−1.08
(A400)	Short stay surgery basic fee	Gamma knife in short stay surgery	0	9874	1797	
M001-3	Radiotherapy with linear accelerator	SRT with linear accelerator	13200	15574	17834	7.81
		Others	2115	3127	4124	18.17
M001-4	Particle therapy	Proton therapy	0	134	2302	314.48
		Heavy particle therapy	0	226	2763	249.65
M002	Total body irradiation	Total body irradiation	921	873	844	−2.16
M003	Hyperthermia therapy	Superficial tumor	552	616	538	−0.64
		Deep-seated tumor	7591	7353	5801	−6.50
Total number of receipts			295813	3007	3217	2.13
				91	88	

Simple^*^: single field and parallel-opposed field radiotherapies Complex^**^: nonparallel-opposed two field and three field radiotherapies, and intracavitary brachytherapy Special^***^: four or more field, rotation, and conformal arc radiotherapies, and interstitial brachytherapy IMRT^****^: intensity-modulated radiation therapy CRPC^*****^: castration-resistant prostate cancer SRT^******^: stereotactic radiotherapy CAGR^*******^: compound annual growth rate N.A. ^********^ : not assessed

The total number of receipts for M000 has also increased slightly at 246,249, 246,505, and 260,491 in 2015, 2017, and 2019, respectively. The ratio of intensity-modulated radiation therapy significantly increased in 2019, compared with that in 2015 (9.5% in 2015 vs. 16.1% in 2019; p < 0.001; Figure 1). The most frequently used radioisotope therapy in 2019 was radioactive iodine treatment for thyroid cancer, followed by radioactive radium treatment for castration-resistant prostate cancer (CRPC) with bone metastases. Radium-223 dichloride, which was administered for patients with CRPC with bone metastases, was mainly used as an alpha-particle emitter.

**Figure 1.**
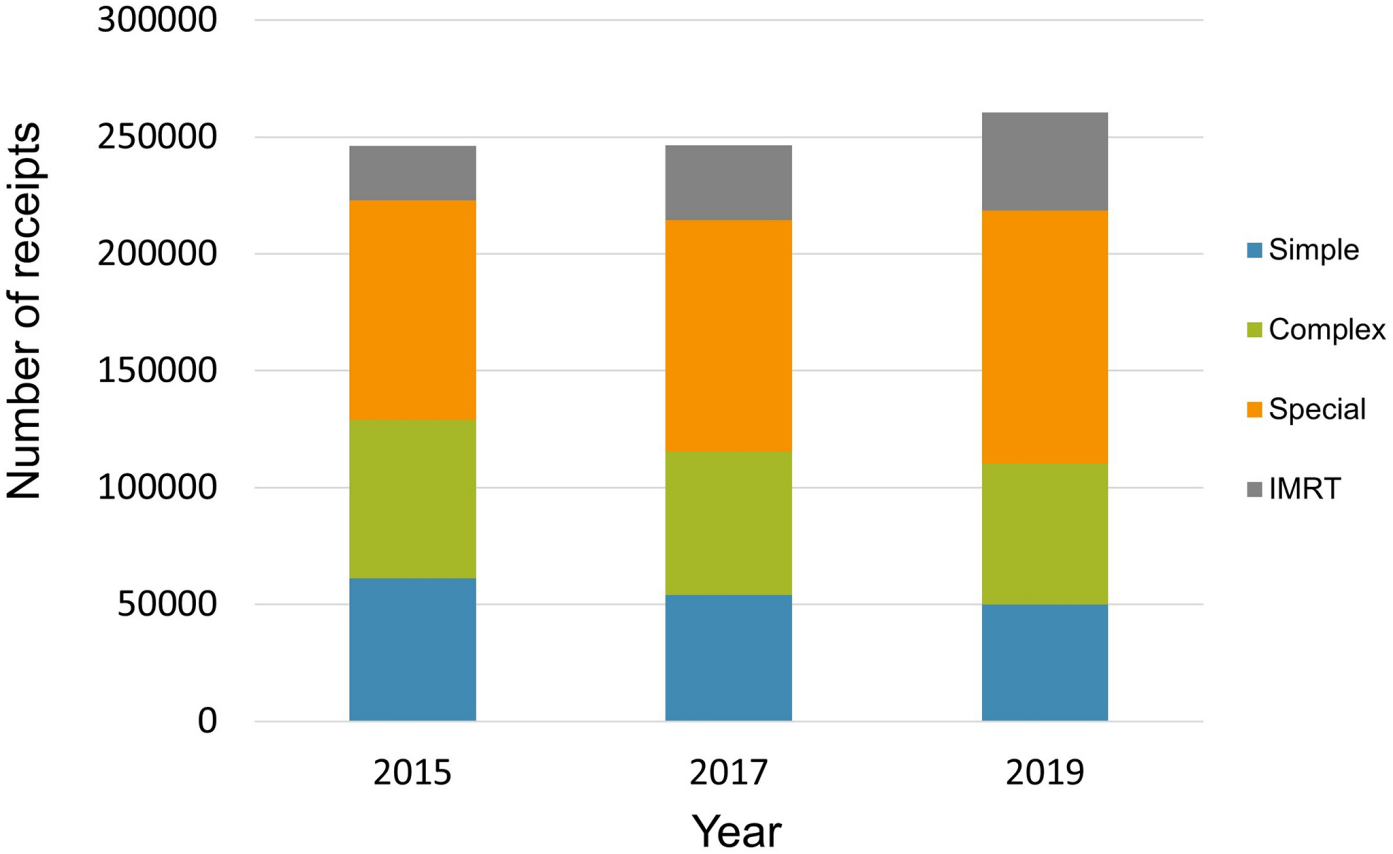
The number of receipts related to medical fee code M000. Simple, single-field and parallel-opposed field radiotherapies; Complex, nonparallel-opposed two-field and three-field radiotherapies and intracavitary brachytherapy; Special, four or more field, rotation, and conformal arc radiotherapies and interstitial brachytherapy; IMRT, intensity-modulated radiation therapy.

The medical fee code from M001-2 to M003 was applied for each one sequence of radiotherapy. The number of receipts associated with particle therapy significantly increased among the radiotherapy modalities from 2017 to 2019 with a CAGR of 275%. The application of stereotactic radiotherapy with a linear accelerator also increased with CAGR of 7.8%. These increases might have been affected by the expansion of type of diseases by national insurance coverage for particle therapy and stereotactic radiotherapy. To compensate for these increases, a decreasing trend was observed for conventional radiation methods. The number of hyperthermia therapy receipts slightly decreased with a CAGR of −6.1% (Figure 2).

**Figure 2.**
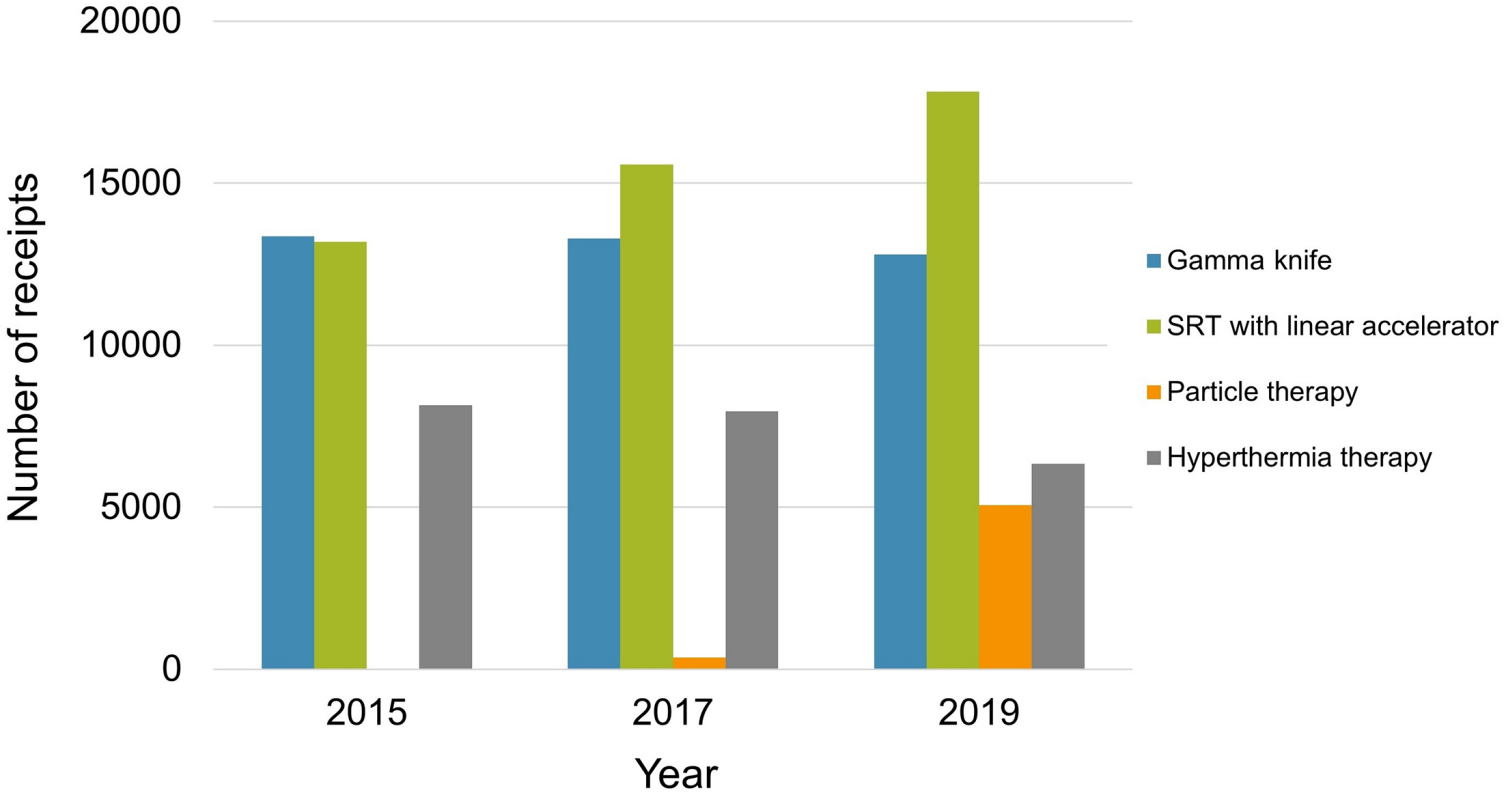
The number of receipts related to medical fee code M001-2 to M003.

In this study, we aimed to conduct an analysis of the national trend of radiotherapy in Japanese patients with cancer based on NDB open data Japan. However, because medical fee codes M000 and M000-2 were calculated multiple times for a single patient, the actual number of patients was approximately lower than the results. According to the most recent version of the Japanese structure survey of radiation oncology, a questionnaire-based survey conducted by the Japanese Society for Radiation Oncology in 2019, the number of patients who received radiotherapy was estimated to be 283,000 ^(7)^.

In 2016-2019, the National Cancer Registry, tabulated by the National Cancer Center Japan, reported approximately 1 million newly diagnosed patients with cancer in the country per year ^(8)^. Although 50%-60% of patients with cancer needed radiotherapy as part of their cancer treatment ^(9), (10), (11)^, <30% of patients with cancer had access to radiotherapy in Japan. This percentage is low compared with that of other developed countries. In South Korea, the percentage of patients who received radiotherapy as the initial treatment increased from 25% in 2010 to 30% in 2015 ^(12)^. In the United States, 31.2% of solid tumors were initially treated with radiotherapy, based on the National Cancer Database ^(13)^. In Japan, only 11.1% of the patients were initially treated with radiotherapy, according to the National Cancer Registry ^(8)^.

This study had several limitations. The receipt data, which were the source of the NDB database, was originally intended for medical fee billing. Thus, the results of this study should be carefully interpreted. Medical procedures were possibly not included if the indication requirements were insufficiently described in the medical records. Additionally, because the data were based on insurance medical receipts, the status of uninsured indications was unclear.

In conclusion, the radiotherapy utilization trend from 2015 to 2019 was determined on the basis of NDB open data Japan. This research was a pilot study, and further investigations on the effect of radiotherapy on various carcinoma types and treatment trends over time, based on NDB, are warranted. The National Cancer Registry reported the radiotherapy utilization rate at initial cancer therapy, not for the entire treatment period. The Japanese structure survey of radiation oncology was a questionnaire-based survey, which has limitations of response rate and unreliability. To compare the National Cancer Registry and the Japanese structure survey of radiation oncology, we considered that NDB is more suitable for analyzing the radiotherapy utilization in Japan. NDB is a comprehensive database for medical insurance in Japan, which has a universal health insurance system. With more than 1 billion receipts added yearly, it is among the largest health-related databases worldwide. The effective use of this database will strongly promote various types of clinical and policy research. With its connection with other public databases in the future, the NDB will hopefully be used for more studies for radiotherapy utilization analysis.

## Article Information

### Conflicts of Interest

None

### Author Contributions

AK wrote the original draft. MM and HY collected and interpreted the clinical data. KN conceived this study and participated in the study design and coordination. HY and KN made substantial contributions to the interpretation of the data. All authors have revised this manuscript critically for intellectual content and have read and approved the final manuscript.

### Approval by Institutional Review Board (IRB)

This study was exempt from institutional review board approval because our study used only anonymized public open data and did not deal with any personally identifiable information.
